# Glutamate transporter: an unexpected target for some antibiotics

**DOI:** 10.1186/1744-8069-1-5

**Published:** 2005-02-09

**Authors:** Jianren Mao

**Affiliations:** 1 Pain Research Group, MGH Pain Center, WACC 324, Massachusetts General Hospital, Harvard Medical School, 15 Parkman Street, Boston, MA 02114, USA

## Abstract

Glutamate transporter (GT) plays a major role in the mechanisms of glutamate homeostasis. Can this transporter system be a therapeutic target for glutamate-mediated neurological disorders? In January's edition of Nature, Rothstein et al (2005) reports that the most commonly used class of antibiotics (β-lactam antibiotics) such as ceftriaxone promoted the expression of GLT1 and demonstrated a functional role in both *in vitro *and *in vivo *models of glutamate neurotocixity. These findings indicate that positive promoters of GT expression may have a unique role in neuroprotection through regulating GT expression. This is also encouraging in search for new pharmacological tools for pain management.

Glutamate is the major excitatory amino acid neurotransmitter that plays an important role in many physiological functions. Maintaining a physiological range of extracellular glutamate concentration is key to preventing glutamate over-excitation and neurotoxicity that could occur under a variety of pathological conditions. Regulating extracellular glutamate is primarily carried out by an efficient, high-capacity glutamate transporter (GT) system, because clearance of extracellular glutamate via glutamate metabolism or diffusion is negligible. To date, at least five cell membrane GT proteins have been cloned [2,3]. GT is labeled by a common name 'excitatory amino acid transporter' (e.g., EAAT1). Among cell membrane GT, EAAT1 (GLAST), EAAT2 (GLT1), and EAAT3 (EAAC1) are particularly relevant to the regulation of glutamate uptake in broad CNS regions. EAAC1 is generally considered as a neuronal GT, whereas GLAST and GLT1 are primarily astroglial GT, although both GLAST and GLT1 also have been located in neuronal cells during the developmental stage [2,3].

Glutamate has a dual role both as an excitatory neurotransmitter essential for physiological functions and a neurotoxic mediator contributory to pathological processes. Since the homeostasis of extracellular glutamate concentration is critically regulated by neuronal and glial GT, reduced GT expression and/or function would be expected to increase extracellular glutamate concentration with subsequent excessive activation of glutamate receptors and excitotoxicity. Indeed, a large number of studies have shown the detrimental effects from reduced GT expression and function on the pathogenesis of neurological disorders including brain ischemia, epilepsy, spinal cord injury, amyotrophic lateral sclerosis, AIDS neuropathy, and Alzheimer's disease.

Given the well-documented role of the glutamatergic system in the mechanisms of pathological pain, it is not surprising that regulation of GT also has been implicated in the central mechanisms of nociceptive processing, such as that following the hindpaw formalin injection or the application of exogenous NMDA or protaglandins in rats [4,5]. A series of recent experiments have demonstrated that the expression of spinal GT was altered following peripheral nerve injury and contributed to neuropathic pain behaviors in rats [6]. The altered GT expression after nerve injury was mediated, at least in part, through a tyrosine kinase receptor (TrkB) and intracellular mitogen-activated protein kinases. Moreover, peripheral nerve injury significantly reduced spinal glutamate uptake activity, supporting a functional role of spinal GT, via regulating regional glutamate homeostasis, in the mechanisms of nerve injury-induced neuropathic pain behaviors [6]. Of interest is that recent studies also have demonstrated that chronic morphine administration regulated the spinal GT expression, which contributed to the mechanisms of morphine tolerance and associated neuronal apoptosis and hyperalgesia in rats [7-9]. Since neuropathic pain and opioid tolerance have been shown to share a common glutamatergic mechanism [10], these findings indicate that regulation of the GT expression and function would be an important approach to preventing and reversing glutamate over-excitation and neurotoxocity associated with the mechanisms of neuropathic pain and opioid tolerance.

Despite a positive regulatory role of certain compounds such as riluzole and MS-153 in GT function in pre-clinical studies of neuropathic pain and morphine tolerance [6-9], the exact mechanisms of GT regulation remain unclear. A recent article by Rothstein et al [1] reports that the most commonly used class of antibiotics (β-lactam antibiotics) such as ceftriaxone promoted the expression of GLT1 and demonstrated a functional role in both *in vitro *and *in vivo *models of glutamate neurotocixity. These findings indicate that positive promoters of GT expression may have a unique role in neuroprotection through regulating GT expression. The regulatory role of β-lactam antibiotics in promoting GT expression appears to be selective, because other classes of antibiotics such as vancomycin were ineffective [1]. The results from this study are encouraging in search for new pharmacological tools for neuroprotection and pain management. A potential issue concerning such an approach, however, is the development of antibiotic-resistant bacteria associated with its application, making it less practical to directly use an antibiotic therapy for managing neuropathic pain, opioid tolerance, and neurological disorders. Therefore, it may be expected that future studies would explore the genomic regulation of GT expression promoted by β-lactam antibiotics in order to elucidate the regulatory pathway(s) of GT expression at the molecular level.

In contrast to antagonism of glutamate receptors, a distinct advantage of regulating GT expression and activity is the possibility of minimizing the pathological impact from a glutamate overload while retaining the physiological role of glutamate. A similar approach is used in developing anti-psychiatric drugs, including broad and selective monoamine reuptake agents, and has yielded rather positive outcomes in the treatment of psychiatric disorders. Extensive research is under way to explore the cellular and molecular mechanisms of GT expression and function in relation to the pathogenesis of neuropathic pain. In addition, studies on the role of GT regulation in opioid tolerance and dependence may provide new insights into the cellular mechanisms of substance abuse, an emerging issue associated with clinical opioid therapy.
